# The influence of bystander presence on evaluations of public breastfeeding among adults in the United States

**DOI:** 10.1186/s12889-023-16635-2

**Published:** 2023-09-09

**Authors:** Amy E. Houlihan, Yuliana Zaikman, Allison M. Alford

**Affiliations:** Department of Psychology & Sociology, Texas A&M University-Corpus Christi, 6300 Ocean Dr., unit 5827, Corpus Christi, TX 78412 USA

**Keywords:** Breastfeeding, Public breastfeeding, Breastfeeding knowledge, Breastfeeding experience, Sexism, Gender

## Abstract

**Background:**

In general, people tend to support private breastfeeding more than public breastfeeding, and discomfort surrounding public breastfeeding may contribute to sub-optimal rates of breastfeeding in the United States. Few studies have systematically examined situational factors that contribute to (negative) reactions to public breastfeeding. It is unclear whether the physical location or the presence of others is more influential in shaping people’s evaluations of public breastfeeding. This study aimed to experimentally investigate the influence of location, bystander presence, bystander gender, and the breastfeeding woman’s use of a cover on people’s evaluations of breastfeeding images.

**Method:**

A sample of adults residing in the United States was randomly assigned to view an image of a breastfeeding woman in an experimental study that examined four independent variables: breastfeeding location (public vs. private), bystander presence (present vs. not present), gender of bystander (male vs. female), and use of a cover (cover vs. no cover). Participants then reported their emotional reactions to, perceptions of, and behavioral intentions toward the breastfeeding woman. In addition, participants completed measures of sexism, traditional gender role endorsement, sexual comfort, body gaze, and breastfeeding knowledge and experience.

**Results:**

Hierarchical regressions revealed no differences between private and public breastfeeding images. Perceptions of the breastfeeding woman were more favorable when she was alone than with others, and when she was covered than when she was not covered. Evaluations tended to be more favorable among participants who scored lower on hostile sexism, higher on benevolent sexism, higher on sexual comfort, and higher on breastfeeding knowledge.

**Conclusion:**

The presence of bystanders may be more consequential than the physical location in shaping reactions to public breastfeeding. These findings can be applied to improve support for public breastfeeding, which may contribute to higher breastfeeding rates and the associated public health benefits.

## Background

Breastfeeding rates in the United States (U.S.) fall well below the recommendations of health agencies such as the World Health Organization and the American Academy of Pediatrics that infants be exclusively breastfed for the first six months of life with continued breastfeeding up to age two [1, 2]. In 2019, only 55.8% of infants in the U.S. were breastfed at six months of age and only 35.9% at 12 months [3]. These low breastfeeding rates are a public health problem, as breastfeeding is associated with lower incidence of respiratory illnesses, type 1 and type 2 diabetes, obesity, and SIDS in infants as well as lower incidence of postpartum depression, breast cancer, ovarian cancer, and type 2 diabetes in women [4–7]. Understanding the factors that contribute to early breastfeeding cessation is critical to improving breastfeeding rates and public health outcomes.

One such factor is perceived social disapproval and negative attitudes toward breastfeeding in public [8–10]. Previous research on evaluations of publicly breastfeeding women is somewhat mixed. Using images of breastfeeding women, some studies have found that privately breastfeeding women are evaluated more favorably than publicly breastfeeding women [11–13]. However, Zaikman and Houlihan [14] found no differences in people’s evaluations based on location. A potential reason for this finding may be that the breastfeeding women depicted in Zaikman and Houlihan’s study were alone, and as such, perhaps participants did not view the breastfeeding as truly “public.” To reconcile these differences, the goal of the current research was to more closely examine situational factors (location, coverage, and bystander presence) as well as observer factors (e.g., breastfeeding knowledge, sexual comfort level) that influence people’s evaluations of breastfeeding women. Based on the overall body of previous research, we predicted that evaluations of a breastfeeding woman alone in a private location would be more favorable than evaluations of a breastfeeding woman in a public location (Hypothesis 1).

### Presence of Bystanders

The presence of bystanders when a woman is breastfeeding can play a role in how the breastfeeding is perceived by observers. When examining people’s perceptions of images of breastfeeding women, Newell and colleagues [15] found that a breastfeeding woman alone was perceived most favorably compared to conditions where other people were present in the background. Similar results were found by Magnusson et al. [13] who argued that levels of proximity and perceived privacy may influence attitudes and perceptions toward breastfeeding in public. In studying real-time reactions to mothers breastfeeding in public locations (e.g., busy city streets, cafes, malls), Grant [16] found that the main factors of influence were the availability of appropriate seating coupled with high privacy or civil inattention from other people in the space. Given previous findings that women who breastfeed in isolation are consistently viewed more favorably than those who breastfeed in the presence of bystanders, we expected that a publicly breastfeeding woman alone will be evaluated more favorably than a publicly breastfeeding woman with a bystander present (Hypothesis 2).

### Bystander gender

In addition to the mere presence of bystanders, the gender of the bystander can also influence evaluations of breastfeeding. Indeed, publicly breastfeeding women surrounded by both men and women were viewed less favorably than publicly breastfeeding women alone or with only one person present [13]. Additionally, mothers who breastfeed around men tend to be viewed less favorably than mothers breastfeeding around other women [17]. When examining transcripts of various family discussions concerning public breastfeeding, Sheehan and colleagues [17] found that the main concern with public breastfeeding was how men would feel around the breastfeeding woman. Men, despite understanding that women had the right to breastfeeding in public, were generally uncomfortable with the topic. Even women were concerned not of their own discomfort as bystanders, but with how men would feel if they had to pass by a breastfeeding woman. This suggests that the perceptions of breastfeeding may stem from concern for the observers, and this concern may be heightened when men are present. This may be due to factors such as sexualization of the breast through the action of “perving” [17] and/or the normalization of the male gaze [16]. In contrast to this pattern of results, Newell and colleagues [15] found that breastfeeding women with female bystanders were viewed less favorably than breastfeeding women with male bystanders. The authors suggested that this finding may be due to confounding variables in the images used, such as the bystander’s proximity to the breastfeeding woman (as the female bystanders were positioned closer to the breastfeeding woman than the male bystanders were). Overall, most research suggests that people are more approving of women breastfeeding around other women than around men. Therefore, it is expected that a breastfeeding woman will be evaluated more favorably when the bystander is female than when the bystander is male (Hypothesis 3).

### Presence of Coverage

Women are often expected to cover themselves when breastfeeding in public because many cultures, Western cultures especially, view the breast primarily as a sexual organ [18]. Sheehan and colleagues [17] suggested that the discomfort stemming from the perceived conflict between women as sexual beings and biologically functioning mothers may be emphasized when observing a woman breastfeeding without coverage. When breastfeeding women are visible to others, there seems to also be a sense of dehumanization of those who do so without coverage, and an assumption that the mothers feel indifferent about the feelings of those around them [17]. However, Zaikman and Houlihan [14] found no difference in perceptions of breastfeeding women who were covered and those who were not. The reason for these findings, as discussed earlier, may be due to the lack of bystanders present in the images of breastfeeding women in Zaikman and Houlihan’s study. Therefore, the current study examined whether evaluations of breastfeeding women differ depending on whether they are wearing a cover (Research Question 1).

### Personal characteristics

Public breastfeeding perceptions and attitudes stem not only from the above situational factors, but also pre-existing characteristics of the observers, which are important to examine alongside the situational factors.

#### Gender

Research findings on gender differences in public breastfeeding attitudes are mixed, with some studies finding no differences [11, 19], others finding that women were more approving [20], and others finding that women were less approving than men [21, 22]. However, given that the most recent similar previous study [14] found that women had more positive perceptions of breastfeeding women than men did (regardless of location), we predicted to replicate that finding in the current study.

#### Sexism

Because breastfeeding is an experience specific to individuals born biologically female (the vast majority of whom identify as women), sexism could influence evaluations of breastfeeding, especially if the action is performed in public and/or without any kind of coverage. Indeed, sexist attitudes tend to play a role in evaluations of breastfeeding [6] and public breastfeeding more specifically [11, 23]. There are two types of sexism as defined by Glick and Fiske [24]. *Hostile sexism* is an overall distrust and dislike of women in general. Those who exhibit hostile sexism typically exhibit more negative thoughts and attitudes toward femininity and womanhood [24]. Conversely, *benevolent sexism* is characterized by idealizing traditional ideas of womanhood. Past research has found that men who have higher levels of benevolent sexism had positive attitudes toward women breastfeeding in private but negative attitudes toward women who perform the same act in public [11]. Additionally, Acker [11] found that people who exhibited higher levels of hostile sexism disapproved of breastfeeding in general, regardless of location and level of privacy. Therefore, we predicted that people exhibiting lower levels of hostile and benevolent sexism will have more favorable evaluations of the publicly breastfeeding woman.

#### Gender role endorsement

Because perceptions of a breastfeeding woman may depend on if she is exhibiting traditional behaviors [11, 24], the endorsement of traditional gender roles may influence these perceptions. Acker [11] suggested a connection between sexist attitudes and one’s endorsement of gender roles in people’s evaluations of public breastfeeding, but past research has scarcely examined this relationship. Olejnik [25] found that people in unmarried romantic relationships had more negative views of public breastfeeding than those who were married. Because those in married relationships are more likely to endorse traditional gender roles [26], we predicted that individuals who adhere more strongly (vs. less strongly) to traditional gender roles would evaluate the publicly breastfeeding woman less favorably overall.

#### Sexual comfort

One’s level of sexual comfort [27] may also influence perceptions of public breastfeeding. Because people with low sexual comfort have less favorable perceptions of breastfeeding in general [14, 28], those negative perceptions may be especially present regarding *public* breastfeeding. Therefore, we predicted that people with higher levels of sexual comfort will exhibit more favorable evaluations of the breastfeeding woman, particularly when the woman is in public and without coverage of the breast.

#### Body gaze

Preoccupation with, and attention paid to, women’s bodies (i.e., body gaze) may underly attitudes toward public breastfeeding. There has been little research studying the effects of body gaze on breastfeeding perceptions. However, given that body gaze is a component of sexual objectification [29], and that sexual objectification as well as sexualization of the breast may influence public breastfeeding perceptions [30, 31], we predicted that those who have higher levels of body gaze will evaluate the breastfeeding woman less favorably than those who have lower levels of body gaze.

#### Breastfeeding knowledge

Understanding the health benefits of breastfeeding and the feeding patterns of infants is likely to predict favorable attitudes toward breastfeeding. Past research found that greater breastfeeding knowledge (acquired through frequent exposure to breastfeeding, educational classes, direct experience, etc.) predicted more favorable evaluations of both private and public breastfeeding [13, 14]. Thus, similarly, we predicted that those possessing more knowledge of breastfeeding will have more favorable evaluations of the breastfeeding woman than those with less knowledge.

#### Breastfeeding experience

Research concerning breastfeeding experience and its relation to breastfeeding perceptions has been fairly consistent. Overall, mothers who breastfed for longer durations tend to have more positive attitudes toward both breastfeeding in private and in public [8, 10]. Therefore, we predicted that those who have experience with breastfeeding will report more favorable evaluations of the breastfeeding woman.

### Overview and hypotheses

The study aimed to experimentally investigate the influence of location, bystander presence, bystander gender, and the woman’s use of a cover on people’s evaluations of breastfeeding images. Our hypotheses and research questions are summarized below. The primary hypotheses refer to predictions regarding variables that were experimentally manipulated, whereas the secondary hypothesis references variables that are characteristics of the participants. We use the term “research question” when we are not making a priori hypothesis for the particular variable of interest. For each hypothesis and research question, evaluations of breastfeeding women are operationalized with four dependent variables: positive emotional responses, negative emotional responses, perceptions, and behavioral intentions.

- Primary Hypothesis 1: Breastfeeding women alone in a private location will be evaluated most favorably compared to women breastfeeding in public locations.
- Primary Hypothesis 2: Breastfeeding women alone in a public location will be evaluated more favorably compared to breastfeeding women in a public location with a bystander present.
- Primary Hypothesis 3: Breastfeeding women in a public location with a female bystander present will be evaluated more favorably compared to breastfeeding women with a male bystander present.
- Research Question 1: Will the use of a cover influence evaluations of breastfeeding women, and will this effect (if any) vary depending on the presence of other people?
- Secondary Hypothesis: Female participants, participants who possess less benevolent and hostile attitudes toward women, less traditional gender roles, greater sexual comfort, lower body gaze, and greater breastfeeding knowledge and experience will have more favorable evaluations of breastfeeding women.

## Methods

### Research design and setting

To test and examine the above hypotheses and research question, a quantitative, experimental design with the following independent variables was utilized: breastfeeding location (public vs. private), bystander presence (present vs. not present), gender of bystander (male vs. female), and use of a cover (cover vs. no cover). The study was conducted with a sample of adults residing in the U.S. recruited through Prolific, an online research platform that connects researchers with participants for online research studies, offering a streamlined process for recruiting and managing participants. The actual administration of the study (including random assignment and collection of data) was conducted via Qualtrics, an online research platform. The study was approved by the Texas A&M University-Corpus Christi Institutional Review Board.

### Participants

An a priori power analysis using G*Power 3.1.9.7 indicated that in order to have statistical power of at least 0.80 for a linear regression, 85 participants are needed to detect a medium sized effect size (*f2* = 0.15), and 600 participants are needed to detect a small sized effect (*f2* = 0.02). Given that Zaikman and Houlihan [14] observed effect sizes ranging from 0.02 to 0.15, we decided to recruit approximately 400 participants. In Fall 2022, a total of 402 U.S. participants were recruited using Prolific. Twenty-seven failed attention check questions (e.g., if you read this, click “strongly agree”), and two participants mentioned photo editing when asked if anything stood out to them about the photo they saw; thus, they were removed from the final sample. Additionally, 16 participants were removed because they failed more than one manipulation check questions (e.g., Who was in the photo you saw?). The final sample included 357 participants (50.7% women) with a median age of 35 (*M* = 38.49, *SD* = 14.29). Most of the sample was White (70.6%), heterosexual (77.9%), agnostic or atheist (44.4%), liberal or very liberal (52.9%), and not parents (59.9%). Participants were compensated $2.10 for their participation.

### Procedure

The anonymized data was collected in September, 2022 and was secured by the second author. Participants were told that the goal of the current study was to examine how people evaluate others. Upon giving their informed consent, they were presented with a randomly assigned photo of a breastfeeding woman. The breastfeeding woman was seated in a private (living room) or public (Starbucks or park) location, alone or with a bystander (who was either male or female), and wearing a breastfeeding cover or not wearing a breastfeeding cover.^1^ The photos of the breastfeeding women alone were the same photos as used in Zaikman and Houlihan [14]. For stimulus sampling purposes [32], the breastfeeding woman in the picture was one of two breastfeeding women used as models. To ensure that we manipulated only the bystander presence in the publicly breastfeeding condition, a professional graphic designer photoshopped the male and female bystanders into the photos of the breastfeeding women in public. The bystanders were depicted walking by the breastfeeding woman from a few feet away and glancing in her direction. In total, participants were assigned to view one of 16 total pictures.

After viewing the photo, participants were asked to rate their emotional responses to the breastfeeding woman, their perceptions of the breastfeeding woman, and their behavioral intentions to interact with the breastfeeding woman. Participants were then asked to complete a series of questionnaires regarding their sexist attitudes, gender role endorsement, sexual comfort, level of body gaze, breastfeeding knowledge, and breastfeeding experience. They then completed a basic demographic questionnaire. Participants were then thanked for their completion of the study.

### Measurement

#### Sexism

Participants completed the short Ambivalent Sexism Inventory [33] consisting of six items assessing hostile sexism, and six items assessing benevolent sexism. Sample items include, “Every man ought to have a woman whom he adores” (benevolent sexism), and “Women exaggerate problems they have at work” (hostile sexism). Participants responded on a scale from 0 (*strongly disagree*) to 5 (*strongly agree*). Average scores were computed for each subscale (α = 0.93 for hostile sexism, α = 0.84 for benevolent sexism).

#### Gender role endorsement

Participants completed the Social Roles Questionnaire [34], a 20-item scale that assessed adherence to gender role attitudes. Sample items include, “A father’s major responsibility is to provide financially for his children” and “Some types of work are just not appropriate for women.” Participants responded to the items on a 5-point scale from 1 (*strongly disagree*) to 5 (*strongly agre*e). An average score was calculated for each participant (α = 0.89).

#### Sexual comfort

Participants’ comfort level with sexual topics was assessed using a modified version of the 20-item Sexual Opinion Survey [35]. Sample items include “I think it would be entertaining to look at erotica (sexually explicit books, movies, etc.)” and “I do not enjoy daydreaming about sexual matters.” (reverse coded). Participants rated their agreement on a 7-point scale ranging from 1 (*strongly disagree)* to 7 (*strongly agree)*. An average score was calculated for each participant (α = 0.92).

#### Body gaze

Participants’ attentional focus on women’s bodies was assessed using five items from the Body Gaze as a Marker of Sexual Objectification scale [29]. Sample items include “Even if a woman’s clothing is not revealing, I still try to look at her body” and “I intentionally position myself to get a better view of the bodies of women.” Participants rated their agreement on a 5-point scale ranging from 1 (*strongly disagree)* to 5 (*strongly agree)*. An average score was calculated for each participant (α = 0.91).

#### Breastfeeding knowledge

Participants’ breastfeeding knowledge was assessed using the 28-item Comprehensive Breastfeeding Knowledge Questionnaire [36]. Sample items included “When a mother is sick with a flu or cold, she should continue to breastfeed her baby as this may prevent her baby from getting sick,” and “The composition of breastmilk changes overtime to meet the needs of the growing baby.” Participants rated their agreement on a 3-point scale with 1 (incorrect), 2 (unsure), and 3 (correct). In order to have correct responses score higher than unsure responses, we recoded the responses as − 1 (incorrect), 0 (unsure), and 1 (correct). Due to a grammatical typo in one of the items (item 13), we removed it from the final scale calculation. A total score was calculated by summing the remaining 27 items for each participant (α = 0.78).

#### Breastfeeding experience

Participants’ personal experience with breastfeeding was assessed with the item, “If you are a parent, was at least one of your children breastfed?” Responses were coded as 0 = no, and 1 = yes (2 = I am not a parent).

#### Emotions

Participants’ positive and negative emotions were measured with the feelings scale [28, 37]. To better capture potential emotional responses to breastfeeding, we also added the following items: *uncomfortable*, *inspired*, *happy*, *interested*, *sad*, and *surprised*, resulting in a total of 18 items. A principal component factor analysis of the 18 items, using varimax rotation, was performed to confirm the two subcategories of the scale and accounted for 52.09% of the variance. The emotions “bored,” “sexually aroused,” and “surprised” loaded weakly, so they were removed from the scales, resulting in a 6-item positive emotion scale and 9-item negative emotions scale. Sample negative emotions include “nauseated,” “angry,” and “uncomfortable,” whereas sample positive emotions include “interested” and “excited.” The emotions were rated on a 6-point Likert scale ranging from 1 (*not at all)* to 6 (*very*). An average score was calculated for each participant (negative emotions α = 0.89; positive emotions α = 0.79).

#### Perceptions

Participants’ perceptions of the breastfeeding woman were measured with the perceptions scale [38], which consists of 20 items rated on from 1 (*strongly disagree)* to 5 (*strongly agree)*. Sample items include, “The seated woman makes a lot of mistakes” (reversed coded), “The seated woman is dependable,” and “The seated woman is respectful.” An average score was calculated for each participant (α = 0.94).

#### Behavioral intentions

Participants’ behavioral intentions to interact with the breastfeeding woman were measured with the 10-item behavioral intentions scale [39]. Sample items include “I want the seated woman as a friend” and “I want to be seen with the seated woman.” Items were rated on a scale from 1 (*not at all)* to 7 (*very much*). An average score was calculated for each participant (α = 0.97).

#### Social desirability

To account for participants’ social desirability bias, the 13-item Marlowe-Crowne Scale [40] was administered. Due to a mistake, only 12 items were included in the survey. Sample items include, “There have been times when I felt like rebelling against people in authority even though I knew they were right” and “I am sometimes irritated by people who ask favors of me.” An average score was calculated for each participant (α = 0.75).

### Data analysis

An independent t-test revealed no significant differences between the two women who were depicted in the photos (positive emotions, *t* = − 0.52, *p* = .61; negative emotions, *t* = − 0.57, *p* = .57; perceptions, *t* = 0.67, *p* = .50; behavioral intentions, *t* = − 0.75, *p* = .45). Therefore, the mother variable was collapsed across the variables of interest.

To examine the hypothesized effects, a set of hierarchical regressions was conducted. The categorical variables were dummy coded such that “female participant,” “covered,” and “parent” conditions were coded 1, and “male participant,” “not covered,” and “not parent” conditions were coded 0. To examine the effect of the location, breastfeeding privately alone was coded as 3, while the other three conditions (publicly breastfeeding alone, publicly breastfeeding with a male bystander, publicly breastfeeding with a female bystander) were coded as − 1. To examine the effect of the presence of others, breastfeeding alone privately was coded as 0, publicly breastfeeding alone was coded as 2, while the other two conditions (publicly breastfeeding with a male bystander, publicly breastfeeding with a female bystander) were coded as − 1. Finally, to examine the effect of the bystander’s gender, breastfeeding alone (privately and publicly) was coded as 0, publicly breastfeeding with a female bystander was coded as 1, and publicly breastfeeding with a male bystander was coded as − 1. Centered versions (based on scale means) of participants’ variables (endorsement of gender roles, hostile sexism, benevolent sexism, sexual comfort, body gaze, breastfeeding knowledge, and social desirability scales) were created. There were no multicollinearity violations with any of the reported results below; all variance inflation factors were under 5 [41].

To examine our hypotheses and research question, the dummy coded variables of location, presence of a bystander, bystander’s gender, and cover were entered in Step 1 All the participants’ variables were centered and entered in Step 1 as well. The hypothesized two-way interactions between the dummy coded variables and cover were entered in Step 2 The above regression was performed four times, once for each of the dependent variables: positive emotions toward the woman, negative emotions toward the woman, perceptions of the woman, and behavioral intentions toward the woman.

## Results

Full results of the regression analyses are presented for each dependent variable in Table 1 (perceptions), Table 2 (behavioral intentions), Table 3 (positive emotions), and Table 4 (negative emotions). Below is a summary of the findings that pertain to our hypotheses.

**Table 1:** Regression analysis of participants’ perceptions of the breastfeeding woman as predicted by manipulated variables and participant variables

Predictor	*β*	*B*	*Se(B)*	*R*^*2*^ Change	95% CI *B*
Step 1				0.28***	
Private vs. Public	0.06	0.02	0.02		[-0.02, 0.07]
Public Alone vs. Public Others	0.11*	0.05	0.02		[0.004, 0.09]
Other Woman vs. Other Man	− 0.03	− 0.02	0.04		[-0.10, 0.05]
Coverage vs. No Coverage	0.10*	0.12	0.06		[0.01, 0.22]
Male Participant vs. Female Participant	0.08	0.09	0.07		[-0.04, 0.22]
Gender Role Endorsement	− 0.06	− 0.002	0.003		[-0.01, 0.003]
Benevolent Sexism	0.25***	0.12	0.03		[0.06, 0.18]
Hostile Sexism	− 0.18*	− 0.08	0.03		[-0.14, − 0.01]
Sexual Comfort	0.23***	0.11	0.03		[0.06, 0.17]
Body Gaze	0.01	0.01	0.03		[-0.06, 0.07]
Breastfeeding Knowledge	0.29	0.03	0.01		[0.02, 0.04]
Social Desirability	0.09†	0.02	0.01		[-0.003, 0.03]
Parent vs. Not Parent	0.01	0.01	0.06		[-0.11, 0.14]
Step 2				0.01	
Private vs. Public * Coverage	0.10	0.06	0.04		[-0.03, 0.14]
Public Alone vs. Public Others * Coverage	− 0.02	− 0.01	0.04		[-0.09, 0.07]
Other Woman vs. Other Man * Coverage	0.08	0.08	0.07		[-0.06, 0.23]
Total *R*^*2*^				0.29	

*N* = 322. *CI *Confidence interval. ‘Male participant,’ ‘No Coverage,’ and ‘Not Parent’ were coded as 0, while ‘Female participant,’ ‘Coverage,’ and ‘Parent’ were coded as 1

†*p* < 0.10; *p < 0.05; **p < 0.01; ***p < 0.001

**Table 2:** Regression analysis of participants’ behavioral intentions toward the breastfeeding woman as predicted by manipulated variables and participant variables

Predictor	*β*	*B*	*Se(B)*	*R*^*2*^ Change	95% CI *B*
Step 1				0.21***	
Private vs. Public	0.04	0.04	0.05		[-0.06, 0.14]
Public Alone vs. Public Others	0.07	0.07	0.05		[-0.03, 0.17]
Other Woman vs. Other Man	− 0.03	− 0.04	0.09		[-0.22, 0.13]
Coverage vs. No Coverage	− 0.01	− 0.04	0.13		[-0.30, 0.23]
Male Participant vs. Female Participant	0.12*	0.32	0.16		[0.004, 0.63]
Gender Role Endorsement	− 0.02	− 0.002	0.006		[-0.01, 0.01]
Benevolent Sexism	0.11†	0.12	0.07		[-0.02, 0.27]
Hostile Sexism	− 0.19*	− 0.18	0.08		[-0.34, − 0.03]
Sexual Comfort	0.19**	0.21	0.07		[0.07, 0.35]
Body Gaze	0.09	0.12	0.08		[-0.04, 0.27]
Breastfeeding Knowledge	0.23***	0.05	0.01		[0.02, 0.07]
Social Desirability	0.15**	0.06	0.02		[0.02, 0.10]
Parent vs. Not Parent	0.02	0.05	0.15		[-0.24, 0.35]
Step 2				0.01	
Private vs. Public * Coverage	0.06	0.08	0.10		[-0.12, 0.28]
Public Alone vs. Public Others * Coverage	− 0.01	0.02	0.10		[-0.18, 0.22]
Other Woman vs. Other Man * Coverage	0.15*	0.38	0.18		[0.03, 0.72]
Total *R*^*2*^				0.23	

*N* = 326. *CI *Confidence interval. ‘Male participant,’ ‘No Coverage,’ and ‘Not Parent’ were coded as 0, while ‘Female participant,’ ‘Coverage,’ and ‘Parent’ were coded as 1

†*p* < 0.10; *p < 0.05; **p < 0.01; ***p < 0.001

**Table 3:** Regression analysis of participants’ positive emotions toward the breastfeeding woman as predicted by manipulated variables and participant variables

Predictor	*β*	*B*	*Se(B)*	*R*^*2*^ Change	95% CI *B*
Step 1				0.14***	
Private vs. Public	0.40	0.03	0.04		[-0.04, 0.10]
Public Alone vs. Public Others	0.07	0.05	0.04		[-0.02, 0.12]
Other Woman vs. Other Man	0.03	0.04	0.06		[-0.08, 0.16]
Coverage vs. No Coverage	− 0.06	− 0.11	0.09		[-0.29, 0.07]
Male Participant vs. Female Participant	− 0.05	− 0.09	0.11		[-0.31, 0.13]
Gender Role Endorsement	− 0.05	− 0.002	0.004		[-0.01, 0.01]
Benevolent Sexism	0.17*	0.13	0.05		[0.03, 0.23]
Hostile Sexism	− 0.001	− 0.001	0.06		[-0.11, 0.11]
Sexual Comfort	0.09	0.06	0.05		[-0.03, 0.16]
Body Gaze	0.18**	0.16	0.06		[0.05, 0.27]
Breastfeeding Knowledge	0.08	0.01	0.01		[-0.01, 0.03]
Social Desirability	0.04	0.01	0.02		[-0.02, 0.04]
Parent vs. Not Parent	0.17**	0.30	0.11		[0.10, 0.51]
Step 2				0.003	
Private vs. Public * Coverage	0.06	− 0.07	0.05		[-0.09, 0.19]
Public Alone vs. Public Others * Coverage	− 0.01	− 0.02	0.05		[-0.15, 0.13]
Other Woman vs. Other Man * Coverage	0.06	− 0.13	0.09		[-0.15, 0.34]
Total *R*^*2*^				0.14	

*N* = 325. *CI* Confidence interval. ‘Male participant,’ ‘No Coverage,’ and ‘Not Parent’ were coded as 0, while ‘Female participant,’ ‘Coverage,’ and ‘Parent’ were coded as 1

†*p* < 0.10; *p < 0.05; **p < 0.01; ***p < 0.001

**Table 4:** Regression analysis of participants’ negative emotions toward the breastfeeding woman as predicted by manipulated variables and participant variables

Predictor	*β*	*B*	*Se(B)*	*R*^*2*^ Change	95% CI *B*
Step 1				0.15***	
Private vs. Public	− 0.005	− 0.002	0.03		[-0.05, 0.05]
Public Alone vs. Public Others	− 0.05	− 0.02	0.03		[-0.07, 0.03]
Other Woman vs. Other Man	0.009	0.007	0.04		[-0.08, 0.09]
Coverage vs. No Coverage	− 0.04	− 0.05	0.07		[-0.18, 0.08]
Male Participant vs. Female Participant	0.04	0.05	0.08		[-0.11, 0.20]
Gender Role Endorsement	0.14	0.004	0.003		[-0.002, 0.01]
Benevolent Sexism	− 0.06	− 0.03	0.04		[0.10, 0.04]
Hostile Sexism	0.10	0.05	0.04		[-0.03, 0.12]
Sexual Comfort	− 0.14*	− 0.07	0.03		[-0.14, − 0.01]
Body Gaze	0.13*	0.08	0.04		[0.004, 0.16]
Breastfeeding Knowledge	− 0.18**	− 0.02	0.01		[-0.03, − 0.01]
Social Desirability	− 0.07	− 0.01	0.01		[-0.03, 0.01]
Parent vs. Not Parent	0.08	0.11	0.07		[-0.04, 0.25]
Step 2				0.01	
Private vs. Public * Coverage	− 0.11	− 0.07	0.05		[-0.16, 0.03]
Public Alone vs. Public Others * Coverage	− 0.03	− 0.02	0.05		[-0.12, 0.08]
Other Woman vs. Other Man * Coverage	− 0.11	− 0.13	0.09		[-0.30, 0.04]
Total *R*^*2*^				0.16	

*N* = 323. *CI* Confidence interval. ‘Male participant,’ ‘No Coverage,’ and ‘Not Parent’ were coded as 0, while ‘Female participant,’ ‘Coverage,’ and ‘Parent’ were coded as 1

†*p* < 0.10; *p < 0.05; **p < 0.01; ***p < 0.001

The first hypothesis was not supported; there were no differences in evaluations of breastfeeding women due to location alone. Our second hypothesis was partially supported. Participants had more favorable perceptions of the woman breastfeeding in public when she was alone than when a bystander was present (*β* = 0.11, *p* < .05, *f2* = 0.02).

**Fig. 1:**
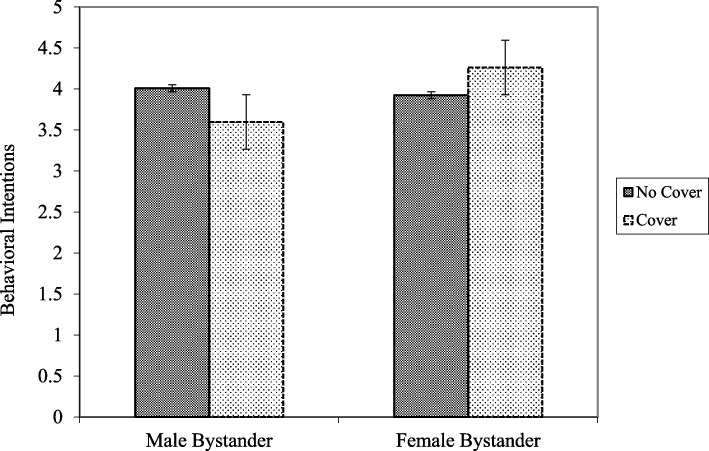
Interaction between bystander gender and cover on behavioral intentions toward the breastfeeding woman

Our third hypothesis was not supported; there were no differences in evaluations of the publicly breastfeeding woman due to the gender of the bystander alone. However, when the use of a cover was considered, there was a two-way interaction between the gender of the other person and the cover (*β* = 0.15, *p* < .05, *f2* = 0.001). Specifically, participants had greater behavioral intentions toward the covered publicly breastfeeding woman when the bystander was a woman compared to when the bystander was a man (*t* = 2.64, *p* < .001; see Fig. 1).

Regarding the research question about the role of a cover, the only effect moderated by the use of the cover was the cover by bystander gender interaction (discussed above). However, there was a main effect of cover for perceptions (*β* = 0.10, *p* < .05, *f2* = 0.009), such that participants had more favorable perceptions of covered (vs. uncovered) breastfeeding women.

Finally, we also examined our secondary hypothesis related to participants’ characteristics. Specifically, we found that female participants had greater behavioral intentions toward the breastfeeding woman compared to male participants (*β* = 0.12, *p* < .05, *f2* = 0.04). Participants who expressed more benevolent sexism toward women had more positive emotions (*β* = 0.17, *p* < .05, *f2* = 0.04) and more favorable perceptions (*β* = 0.25, *p* < .001, *f2* = 0.004). Additionally, participants who expressed more hostile sexism toward women had less favorable perceptions (*β* = − 0.18, *p* < .05, *f2* = 0.06) and lower behavioral intentions toward the woman (*β* = − 0.19, *p* < .05, *f2* = 0.07). Participants who were more sexually comfortable had fewer negative emotions (*β* = − 0.14, *p* < .05, *f2* = 0.04), more favorable perceptions (*β* = 0.23, *p* < .001, *f2* = 0.08), and greater behavioral intentions toward the woman (*β* = 0.19, *p* < .01, *f2* = 0.06). Participants who were more likely to engage in body gaze had more negative emotions (*β* = 0.14, *p* < .05, *f2* = 0.03) and more positive emotions (*β* = 0.18, *p* < .01, *f2* = 0.06). Additionally, participants who were more knowledgeable about breastfeeding had fewer negative emotions (*β* = − 0.18, *p* < .01, *f2* = 0.05), more positive emotions (*β* = 0.14, *p* < .05, *f2* = 0.01), more favorable perceptions (*β* = 0.29, *p* < .001, *f2* = 0.16), and greater behavioral intentions toward the woman (*β* = 0.23, *p* < .001, *f2* = 0.11). Participants who were parents had more positive emotions (*β* = 0.17, *p* < .01, *f2* = 0.04). Finally, participants’ endorsement of gender roles did not influence their evaluations of breastfeeding women. Therefore, our secondary hypothesis regarding personal characteristics was partially supported.

To examine breastfeeding experience more closely, we selected the subsample of participants who reported being parents and computed the above stated analyses again, substituting the parent status variable for the breastfed child variable. The subsample included 143 participants (60.8% women) with a median age of 46 (*M* = 46.88, *SD* = 14.47). Parents who were more likely to engage in body gaze had more negative emotions (*β* = 0.26, *p* < .05, *f2* = 0.04) and more positive emotions (*β* = 0.27, *p* < .05, *f2* = 0.11) in response to the breastfeeding woman. Moreover, parents who were more sexually comfortable had more favorable perceptions (*β* = 0.28, *p* < .01, *f2* = 0.15), and greater behavioral intentions toward the woman (*β* = 0.31, *p* < .01, *f2* = 0.14). Finally, parents who were more knowledgeable about breastfeeding had more favorable perceptions (*β* = 0.33, *p* < .001, *f2* = 0.19), and greater behavioral intentions toward the woman (*β* = 0.23, *p* < .05, *f2* = 0.14).

## Discussion

In contrast to older research [11, 13] but consistent with previous recent research [14], evaluations of privately breastfeeding women did not differ significantly from those of publicly breastfeeding women; thus, the physical location of the breastfeeding was not influential. Instead, results of the current study suggest that people are sensitive to the presence or absence of bystanders when evaluating public breastfeeding. Consistent with Newell et al. [15], women breastfeeding in public locations were evaluated less favorably when others were present than when they were alone. Thus, the “public” nature of the public breastfeeding appears not to be driven by the location per se, but rather by the presence of others.

Somewhat surprisingly, the gender of the bystander did not appear to directly influence evaluations; however, behavioral intentions were highest when the breastfeeding woman was covered with a female bystander compared to a male bystander. This suggests that even when the breastfeeding woman is covered, people are not especially comfortable with women breastfeeding around men. Support for this explanation comes from an informal analysis of qualitative responses to a question about whether the participants thought there was anything weird or odd about the photo they viewed (the purpose of this question was to identify participants who perceived that the photos were digitally altered). Several comments were made in reference to the male bystander “staring” or “ogling” the breastfeeding woman (these types of comments were not made about the female bystander; in fact, the female bystander was never specifically mentioned). Although anecdotal, this qualitative data suggests that at least some participants were uncomfortable with a man observing a woman breastfeeding in public, and that the use of a cover did not necessarily eliminate their concerns. However, additional research is necessary to further examine how bystander gender and the presence/absence of a cover influence evaluations of public breastfeeding.

To our knowledge, this is the first study that systematically manipulated the presence (and gender) of bystanders in photographs of the same breastfeeding women in the same locations. Although others [12, 13, 15] have examined reactions to images of public breastfeeding, the lack of standardization across photographs (resulting in confounding factors, including the woman’s expression and body position, amount of breast exposure, proximity to bystanders, age of child) limited the ability to draw conclusions about the role of each factor in shaping evaluations of public breastfeeding. In contrast, we can be more certain that the differences observed in the current study are due to the variables of interest. In addition, the current study is one of the only studies to experimentally examine the influence of the use of a cover in evaluations of public breastfeeding. The finding that perceptions were more favorable when the woman was covered vs. uncovered (regardless of location or bystander presence) suggests that exposure of the breast may be central to people’s objections to public breastfeeding. Thus, the current study expands and improves upon the limitations of previous research and provides a foundation for additional future studies to examine these variables more extensively.

In addition to the effects of the experimentally manipulated variables, the current study also found that participant characteristics influenced evaluations of public breastfeeding in ways that were largely consistent with previous research [14]. Gender-related factors (participant gender, benevolent and hostile sexism), sex-related factors (sexual comfort and body gaze), and breastfeeding knowledge were associated with evaluations of breastfeeding (regardless of location, bystander presence, and bystander gender). Breastfeeding knowledge in particular was most consistently associated with evaluations of publicly breastfeeding women (it was significantly associated with all four dependent measures). This highlights the need for breastfeeding education among the general population (parents and non-parents alike), as our results show that breastfeeding knowledge may play a greater role in shaping evaluations of public breastfeeding than does direct experience (i.e., having a child who was breastfed). When examining only the sub-sample of parents, having a child who was breastfed was not significantly associated with evaluations of breastfeeding (this is in contrast to the findings of Zaikman and Houlihan [14], but the discrepancy is likely due to the slightly different analysis performed in which breastfeeding experience was analyzed separately from other participant characteristics). It is clear from these findings that evaluations of breastfeeding women are not based solely on situational factors; observers bring a wide range of pre-existing attitudes and beliefs to the situation. Future research on public breastfeeding should continue to identify and explore participant characteristics, especially as they relate to or interact with situational factors.

### Limitations and future directions

Limitations of the current study include the limited generalizability of our findings due to the use of an American sample that was predominantly White. Breastfeeding attitudes and practices vary by ethnicity, culture, and socioecological context [42–44], so it should not be assumed that our findings reflect responses to public breastfeeding among other populations. Future research should replicate this study with more diverse samples of Americans as well as with samples from other countries so that cross-cultural comparisons in evaluations of public breastfeeding can be made. Additionally, the study examined participants’ evaluations of single photographs, which has limited mundane realism. The study may be strengthened by the use of videos (or even live scenarios) of public breastfeeding that would capture the action of breastfeeding more dynamically and naturally than still photographs. Furthermore, participants viewed the photographs from an “outside” perspective, meaning they were not asked to imagine that they were present in the scenario they viewed. It is possible that asking participants to imagine themselves in the position of the bystander may elicit different responses to the public breastfeeding, but this remains to be tested in future research.

Future directions also include replicating the current study with the manipulation of additional factors, including the number of bystanders present, the proximity of the bystander(s) to the breastfeeding woman, and the specific activities taking place in the situation (e.g., dining in a restaurant, working in an office, shopping in a store). These factors may influence reactions to public breastfeeding but have largely been unexamined in the extant literature. In addition, although we attempted to account for as many relevant participant characteristics as possible, there may be additional characteristics that influence evaluations of public breastfeeding (e.g., religiosity). Lastly, the results of this study may inform the development of interventions designed to promote breastfeeding or increase support for public breastfeeding; for example, given the influential role of breastfeeding knowledge, interventions designed to educate people about breastfeeding in general (or public breastfeeding in particular) may improve evaluations of public breastfeeding.

## Conclusions

Using an experimental design, the current study found that the presence of bystanders and the use of a cover influence people’s evaluations of publicly breastfeeding women. The presence of bystanders was more consequential than the actual physical location in shaping reactions to public breastfeeding, and this construct (presence of others) should be considered when future researchers seek to operationalize “public” breastfeeding. Additionally, people’s pre-existing characteristics including gender, sexist attitudes, sexual comfort, body gaze, and breastfeeding knowledge influence their evaluations of public breastfeeding. These findings may be used in the future to strengthen support for women in breastfeeding in public, which may result in higher breastfeeding rates and improved public health.

## Data Availability

The dataset used and analyzed during the current study are available from the corresponding author on reasonable request.

## Footnotes

1 The number of participants assigned to each condition are as follows: private location breastfeeding alone with no cover (n = 23), private location breastfeeding alone with cover (n = 25), public location breastfeeding alone no cover (n = 51), public location breastfeeding alone with cover (n = 51), public location breastfeeding with male bystander no cover (n = 46), public location breastfeeding with female bystander no cover (n = 55), public location breastfeeding with male bystander with cover (n = 58), public location breastfeeding with female bystander with cover (n = 48).
